# Reduced extrinsic recombination process in anatase and rutile TiO_2_ epitaxial thin films for efficient electron transport layers

**DOI:** 10.1038/s41598-021-86422-9

**Published:** 2021-03-24

**Authors:** Yeon Soo Kim, Hye-Jin Jin, Hye Ri Jung, Jihyun Kim, Bich Phuong Nguyen, Juran Kim, William Jo

**Affiliations:** Department of Physics and New and Renewable Energy Research Center (NREC), Ewha Womans University, Seoul, 03760 Korea

**Keywords:** Solar cells, Materials for energy and catalysis

## Abstract

TiO_2_ is the most widely used material for the electron transport layers (ETLs) because it is characterized by proper band alignment with light absorbers, adequate optical transmittance, and high electron mobility. There are two thermodynamically stable crystal phases of TiO_2_: anatase and rutile. However, understanding which phase is more effective as the ETL is still required. In this paper, we demonstrate the different effects of using epitaxial anatase TiO_2_ and epitaxial rutile TiO_2_ (both grown using pulsed laser deposition) as the ETL material on the electrical and optical properties. Epitaxial Nb-doped TiO_2_ layers were used as the common electrode material for the both epitaxial ETLs for which the crystalline structural analysis revealed high crystalline qualities and good coherency for both phases. By analyzing the recombination kinetics, the anatase phase shows a preferable performance in comparison with the rutile phase, although both epitaxial phases show remarkably reduced extrinsic recombination properties, such as trap-assisted recombination. This study demonstrates not only a better electron transporting performance of anatase phase but also reduced extrinsic recombination through epitaxy growth.

## Introduction

TiO_2_ is a wide band gap semiconductor, one of the most extensively used transition metal oxide materials due to its various applications in, for example, light-emitting diodes^1^, photocatalysts^2^, solar cell conversion devices^3^, high-*k* gate insulators^4^, and waveguides^5^. These various applications are strongly dependent on the different characteristics associated with each of the three crystal phases of TiO_2_: anatase, rutile, and brookite. However, because the brookite polymorph is the most unstable phase due to its complex structure^6^, most of the practical applications and research involve the thermodynamically stable anatase and rutile phases. There have been numerous studies on the comparison between the anatase and rutile phase, including those regarding the optical properties^7^ and photocatalytic activity^2^ so far. In addition, it is well-known that the TiO_2_ satisfies most of conditions for the solar cell application, which having proper band alignment with the solar cell absorber layers for the efficient transporting of electrons and blocking of holes, adequate optical transmittance as well as high electron mobility to extract carriers from the light absorber layer and avoid charge recombination^6^.

Although TiO_2_ is currently the most commonly used electron transport layer (ETL) material in most of the solar cell applications, however, there is still debate as to whether the anatase^2,8^ or the rutile^9,10^ phase is more effective as an ETL in the solar cell application, such as dye-sensitized solar cells (DSSCs) and halide perovskite solar cells (HPSCs). The main reason for this debate is that the formation of the crystalline phase is not performed in a manner similar to that of a single crystal because most TiO_2_ ETLs are deposited by spin coating, pyrolysis, or sputtering, which is advantageous for large-area fabrication of thin films. As a result, there is a lack of research on the difference between the anatase phase and the rutile phase, and understanding this difference could minimize the various defects formed in the ETL, such as oxygen vacancies and grain boundaries due to an amorphous-like polycrystalline structure. In particular, a factor of which a direct comparison could prevent the occurrence of these various defects in the anatase and rutile phase is trap-assisted recombination. Extrinsic recombination losses cause an unexpected open-circuit voltage drop that lowers the fill factor^11^ and the resulting trap center affects the long-term stability that causes the defect-induced degradation of the solar cell^12^. Therefore, a nearly defect-free single crystalline phase of anatase and rutile TiO_2_ thin films are a prerequisite.

In this study, the single crystalline phase of anatase and rutile thin films were fabricated using pulsed laser deposition, which is disadvantageous for fabricating large-area thin films but provides excellent control of the growth condition to obtain a high-quality thin film. High-resolution X-ray diffraction (HR-XRD) and high-resolution transmission electron microscopy (HR-TEM) measurements show high crystalline qualities of anatase and rutile epitaxial thin films.

## Results and discussion

**Figure 1:**
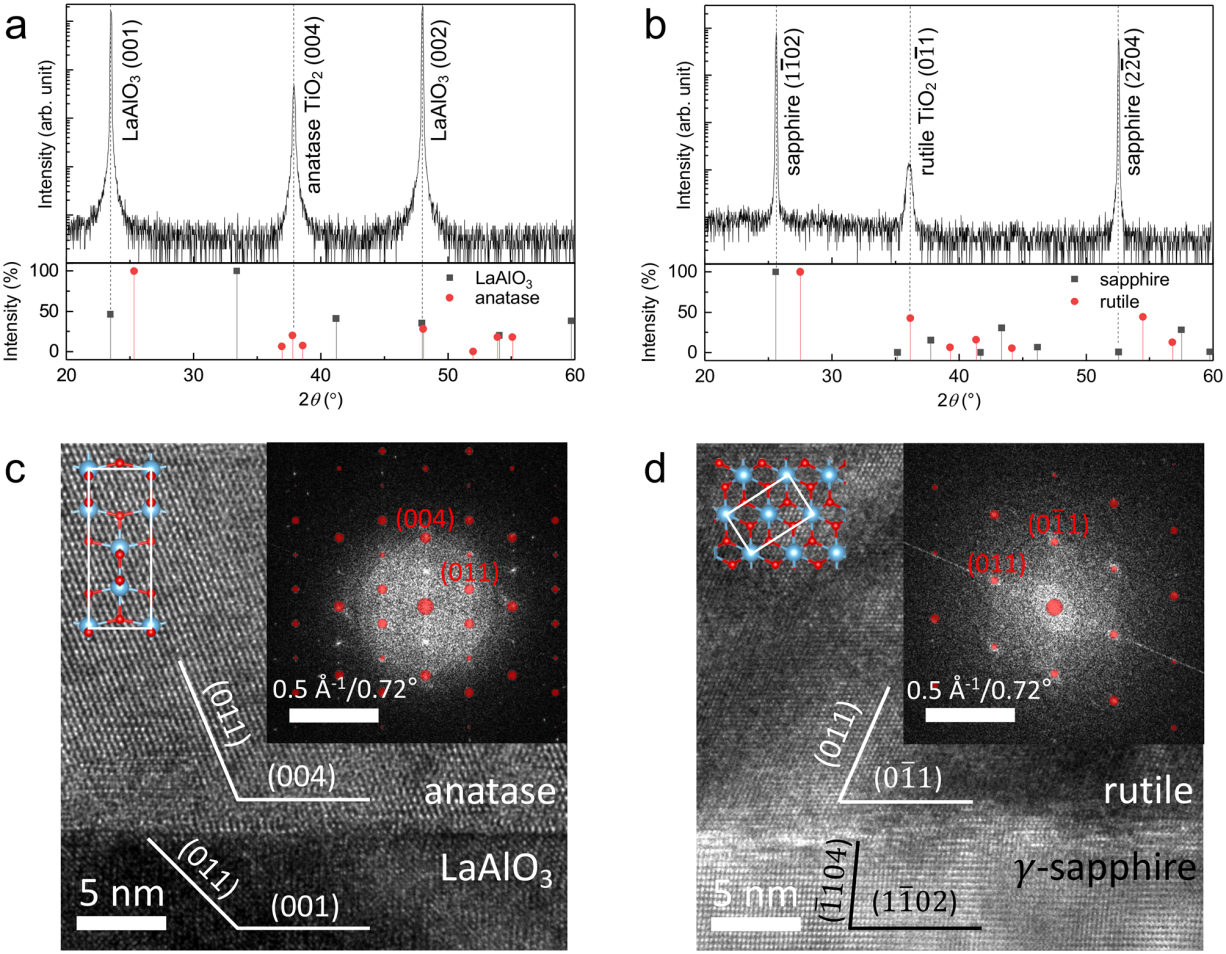
Crystallographic properties of epitaxial anatase and rutile TiO_2_ thin films. X-ray diffraction data of (**a**) epitaxial anatase TiO_2_ thin film/LaAlO_3_ and (**b**) rutile TiO_2_ thin film/γ-sapphire (top) with corresponding International Centre for Diffraction Data **(**ICDD; bottom, LaAlO_3_, ICDD 98-017-0772; anatase TiO_2_, ICSD 98-000-9852; sapphire, ICDD 01-089-3072; rutile TiO_2_, ICDD 03-065-0192). Cross-sectional HRTEM images of: (**c**) anatase TiO_2_ thin film/LaAlO_3_ and (**d**) rutile TiO_2_ thin film/γ-sapphire. The insets of (**c**) and (**d**) (top left) show crystal structure of the anatase and the rutile phases, respectively. White rectangles represent a unit cell. The insets of (**c**) and (**d**) (top right) show the FFT patterns and simulated results (red circle) of the anatase TiO_2_ thin film and rutile TiO_2_ thin film, respectively.

Figure 1a,b shows the X-ray diffraction (XRD) pattern of epitaxial TiO_2_ thin films grown on LaAlO_3_ (001) and γ-sapphire (1$\overline{1}$02) single crystal substrates, respectively. The XRD results demonstrate that there is no secondary phase except for the anatase and rutile phase on the LaAlO_3_ and γ-sapphire substrates, respectively. The lattice parameter *c* for the anatase TiO_2_ thin film on the LaAlO_3_ substrate is 9.495 Å, which is only a -0.2% lattice mismatch with bulk anatase TiO_2_ (9.514 Å). Additionally, the lattice spacing in the [011] direction of epitaxial rutile TiO_2_ thin film on the γ-sapphire substrate is 2.489 Å, which is very close to the 2.487 Å lattice spacing of bulk rutile TiO_2_. The thickness of both films was measured by X-ray reflectometry to be approximately 40 nm (Fig. S1, Supplementary Information). Figure 1c,d shows the cross-sectional HR-TEM images of anatase TiO_2_ thin film/LaAlO_3_ and rutile TiO_2_ thin film/γ-sapphire, respectively. Both phases show clearly observable crystal structures and interfaces between the substrates. The crystal orientations were determined to be anatase (004) // LaAlO_3_ (001) and rutile (0$\overline{1}$1) // γ-sapphire (1$\overline{1}$02) by their corresponding fast Fourier transform (FFT) patterns shown in the insets of Fig. 1c,d, respectively. The half-transparent red circles denote simulated diffraction pattern results. The estimated lattice spacings of anatase (100), (001), and (110) from the TEM images are 3.928 Å, 9.077 Å, and 3.571 Å, respectively. These values are slightly different from the lattice spacings of bulk anatase, which are 3.785 Å, 9.514 Å, and 3.517 Å, respectively. This difference may be a result of the tensile strain in the vicinity of the interface due to the difference in the in-plane lattice parameter between the anatase (a = 3.785 Å) and LaAlO_3_ (a = 3.821 Å) substrate. Taking into consideration the relaxation of the anatase thin film corresponding to the substrate as estimated from the TEM images, the lattice spacings of anatase {100}, {001}, and {110} were adjusted to 3.872 Å, 9.089 Å, and 3.562 Å, respectively, values of which are more comparable to those of the TEM results. This implies that the anatase thin film has in-plane coherency with the substrate. The lattice spacings in rutile (0$\overline{1}$1) and (011) obtained from the TEM image are 2.530 Å and 2.501 Å, which resulted in lattice distortions from the lattice spacings of bulk rutile, which are 2.487 Å and 2.487 Å, respectively (Fig. S4, Supplementary Information). Detailed information about the lattice distortion is referred in Table S1 in Supplementary Information.

For the electrical measurement of TiO_2_ thin films along the out-of-plane direction, the conductivity of the bottom electrode on the substrate is essential. The bottom electrode should have a small lattice mismatch with both the substrate and TiO_2_ thin film for the epitaxial growth of the TiO_2_ thin film; however, it is difficult to find a suitable electrode material with a small lattice mismatch with both the LaAlO_3_ (001) and γ-sapphire (1$\overline{1}$02) substrates. Several perovskite oxides are suitable as good electrode material on LaAlO_3_ substrates, such as La_0.5_Sr_0.5_CoO_3_^7^ and LaNiO_3_^13^, however, they have a large lattice mismatch with γ-sapphire substrates. In contrast, binary oxides, such as RuO_2_^7^, have a small lattice mismatch with γ-sapphire substrates, but epitaxial growth on LaAlO_3_ substrates is difficult. Furthermore, using different electrodes on different substrates would hinder the direct comparison of the optoelectronic properties between anatase and rutile TiO_2_ thin films. The addition of dopants, however, may be an effective strategy. In previous studies, transition metal ions, such as Nb and Y, were used as dopants in TiO_2_ to produce efficient ETLs^14,15^. In this study, 10 wt% Nb-doped TiO_2_ target was prepared as bottom electrodes.

**Figure 2:**
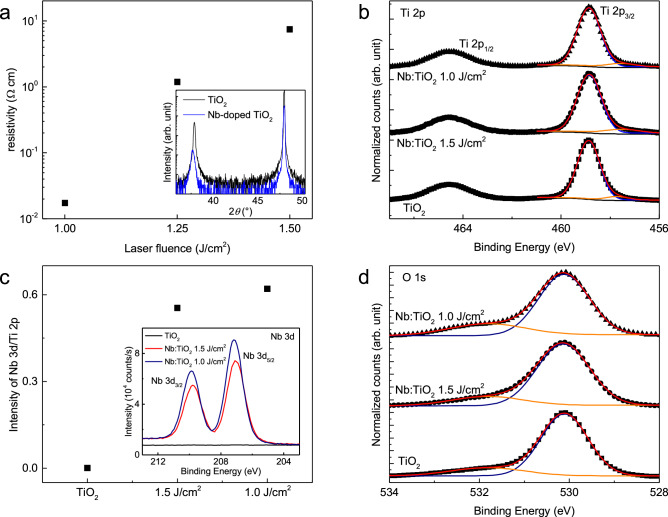
Characteristics of anatase Nb-doped TiO_2_ epitaxial thin film. (**a**) Resistivity of anatase Nb-doped TiO_2_ thin films as a function of the laser fluence. Inset of (**a**) shows the XRD patterns of anatase TiO_2_ and Nb-doped TiO_2_ thin films. (**b**) XPS spectra of Ti 2p of anatase TiO_2_ and Nb-doped TiO_2_ thin films with varying laser fluence. The measured spectra of the laser-fluence-dependent Ti 2p_3/2_ (black dots) deconvolved into the characteristic Ti^4+^ peaks (blue), Ti^3+^ peaks (orange), and summation of the peaks (red). (**c**) Ratio intensity of Nb 3d peaks and Ti 2p peaks with varying laser fluence. Inset of (**c**) represents the XPS spectra of Nb 3d for anatase TiO_2_ and Nb-doped TiO_2_ thin films. (**d**) XPS spectra of O 1 s for anatase TiO_2_ and Nb-doped TiO_2_ thin films with varying laser fluence. The measured spectra of O 1 s (black dots) deconvolved into the characteristic lattice oxygen peaks (blue), non-lattice oxygen peaks (orange), and the summation of the peaks (red).

Figure 2a shows the resistivity as a function of laser fluence varying from 1.0 to 1.5 J cm^−2^, with a lower laser fluence leading to more metallic films. In a previous study^16^, increasing laser fluence lead to the preferential ablation of lighter atoms, or Ti atoms in this case, whereas decreasing laser fluence resulted in relatively Nb-abundant thin films. Additionally, the XRD pattern in the inset of Fig. 2a suggests that the diffraction peak of the Nb-doped TiO_2_ (004) is located at a lower angle than that of the TiO_2_ (004) peak. This peak shift indicates a lattice expansion of the Nb-doped TiO_2_ thin film of approximately 0.5%. The lattice expansion is observed in rutile Nb-doped TiO_2_ thin film/γ-sapphire as well. (Fig. S3, Supplementary information) There are a couple of reasons for the lattice expansion associated with the Nb dopants: a larger effective ionic radius of Nb^5+^ (78 pm) compared with Ti^4+^ (74.5 pm)^17^ and a resulting Coulomb repulsion due to the donor ion^18^.

The X-ray photoelectron spectroscopy (XPS) spectra of Ti 2p for the anatase TiO_2_ and Nb-doped TiO_2_ thin films are shown in Fig. 2b. The Ti 2p_3/2_ and Ti 2p_1/2_ peaks are located at the binding energies of 458.9 eV and 464.5 eV, respectively, which arise mainly from the Ti^4+^ in the TiO_2_ lattice^19^. Additionally, the XPS results of the Ti 2p spectra exhibit broad peaks at 457.4 eV, denoting the existence of a small quantity of Ti^3+^ species. In contrast to a previous study^20^, as Nb contents increased, the percentage of peak area for Ti^3+^ slightly decreased at 4.31%, 4.15%, and 3.86% for TiO_2_, Nb-doped TiO_2_ with a laser fluence of 1.5 J cm^−2^, and Nb-doped TiO_2_ with a laser fluence of 1.0 J cm^−2^, respectively (Fig. S5, Supplementary Information). Figure 2c shows that as the laser fluence increases, the intensity of Nb 3d decreases. Similarly, both the intensity of the Nb 3d peak as well as the intensity ratio of Nb 3d_5/3_ to Ti 2p_3/2_ increase as the laser fluence decreases (see inset of Fig. 2c). These results imply that the resistivity of the Nb-doped TiO_2_ thin film can be controlled with stoichiometric modulation by varying the intensity of the laser fluence. Figure 2d shows the O 1 s spectra of TiO_2_, Nb-doped TiO_2_ with a laser fluence of 1.5 J cm^−2^, and Nb-doped TiO_2_ with a laser fluence of 1.0 J cm^-2^. The peaks at the binding energies of 530.1 eV and 531.8 eV are attributed to the lattice oxygen and hydroxyl groups, or non-lattice oxygen, respectively^19^. The increase in the area of the non-lattice oxygen with the increase in Nb dopants, as shown in Fig. S4, is due to the increase in the formation energy of the oxygen vacancies due to the lattice deformation induced by the Nb dopants^21^ and/or the low Gibbs free energy of oxidation of Nb and not Ti^22^. Using anatase Nb-doped TiO_2_ epitaxial thin films and rutile Nb-doped TiO_2_ epitaxial thin films, both with a 1 J cm^−2^ laser fluence, as a bottom electrode on the LaAlO_3_ and γ-sapphire substrate, respectively, the 20-nm-thick anatase and rutile TiO_2_ thin films were deposited as ETLs. Furthermore, the anatase Nb-doped TiO_2_ epitaxial thin films and rutile Nb-doped TiO_2_ epitaxial thin films, both with a 1.5 J cm^−2^ laser fluence, were also used as ETLs because using dopants such as Nb can improve carrier extraction or injection^23^.

**Figure 3:**
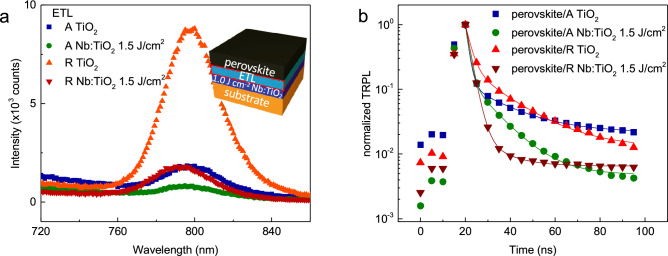
PL and TRPL results. A and R denote the anatase and the rutile phases respectively. (**a**) Steady-state PL spectra. (**b**) Normalized TRPL spectra of perovskite films on various ETLs. Lines indicate double exponential fitting results. A schematic of the HPSC is shown in the inset of (**a**).

Owing to their tunable band gap^3^, long carrier diffusion length^24^, high optical absorption coefficient^25^, and easy fabrication process at low temperature^3^, organometal halide perovskites have attracted much attention as very promising solar cell absorbers^3^, photodetector^26^, light-emitting diode^27^, field-effect transistor^28^ and memory elements^29^. Thus, the HPSC provides a great platform to investigate charge transporting and dynamics in the photovoltaic applications. The conventional architecture of HPSCs consists of cathode/ETL/perovskite/hole transport layer (HTL)/anode. We prepared organometal halide perovskites as a light absorber layer, for which details regarding the fabrication method are described in the Methods section. To investigate charge dynamics, we obtained measurements for the steady-state photoluminescence (PL) and time-resolved PL (TRPL) upon excitation at 405 nm. Figure 3a shows the steady-state PL spectra of the HPSCs with the different types of substrates. The schematic for the device structure is depicted in the inset of Fig. 3a. It can be observed that the PL peak considerably quenches in the HPSC with the ETL consisting of the anatase thin film in comparison with the other types of HPSC, signifying that using TiO_2_ in the anatase phase could provide better electron transport and charge separation compared with using TiO_2_ in the rutile phase. In addition, Nb-doped TiO_2_ thin films as ETLs with either the anatase or rutile phase are more effective than undoped TiO_2_ thin films. Figure 3b shows the normalized TRPL for the various substrates measured at an emission wavelength of about 800 nm. A bi-exponential decay fitting was performed and can be expressed as

1 $$I = A_{1} exp\left( { - t/\tau_{1} } \right) + A_{2} exp\left( { - t/\tau_{2} } \right),$$

where τ_1_ and τ_2_ denote the time for the slow delayed recombination at the trap center and the bimolecular recombination, which occurred on a relatively short time scale, respectively^30^. The delayed recombination component and the bimolecular recombination component of the anatase TiO_2_ thin film was 17.83 ns and 1.45 ns, respectively, whereas the delayed and bimolecular recombination was 16.35 ns and 2.37 ns for the rutile TiO_2_ thin films, respectively. It can be observed that the amplitude of the bimolecular decay component is much larger than that of the slow decay (Table 1). The near absence of non-radiative recombination gives rise to the shortest reported average decay component, which can be expressed as $\tau_{AVG} = \mathop \sum \limits_{i} A_{i} \tau_{i}^{2} /\mathop \sum \limits_{i} A_{i} \tau_{i}$. This implies that electrons are effectively extracted from the absorber layer to the ETL without significant non-radiative recombination effect by minimizing undesirable trap sites inside the bulk, particularly more so in the anatase phase rather than in the rutile phase. Interestingly, the bimolecular decay component of anatase and rutile Nb-doped TiO_2_ epitaxial thin films as ETLs is comparable to that of anatase and rutile TiO_2_ epitaxial thin films. Thus, the effects due to the phase difference between the anatase phase and the rutile phase appear to be more dominant than the effects due to the addition of Nb dopants.

**Table 1:** Parameters of the TRPL spectra of perovskite on different ETLs.

ETL	A_1_	*τ*_1_ (ns)	A_2_	*τ*_2_ (ns)	A_2_/(A_1_ + A_2_)
A TiO_2_	0.30	17.83	851,342	1.45	1
A 1.5 J/cm^2^	0.86	10.99	394,778	1.53	1
R TiO_2_	0.70	16.35	3642	2.37	0.99981
R 1.5 J/cm^2^	0.12	10.66	6460	2.27	0.99998

**Figure 4:**
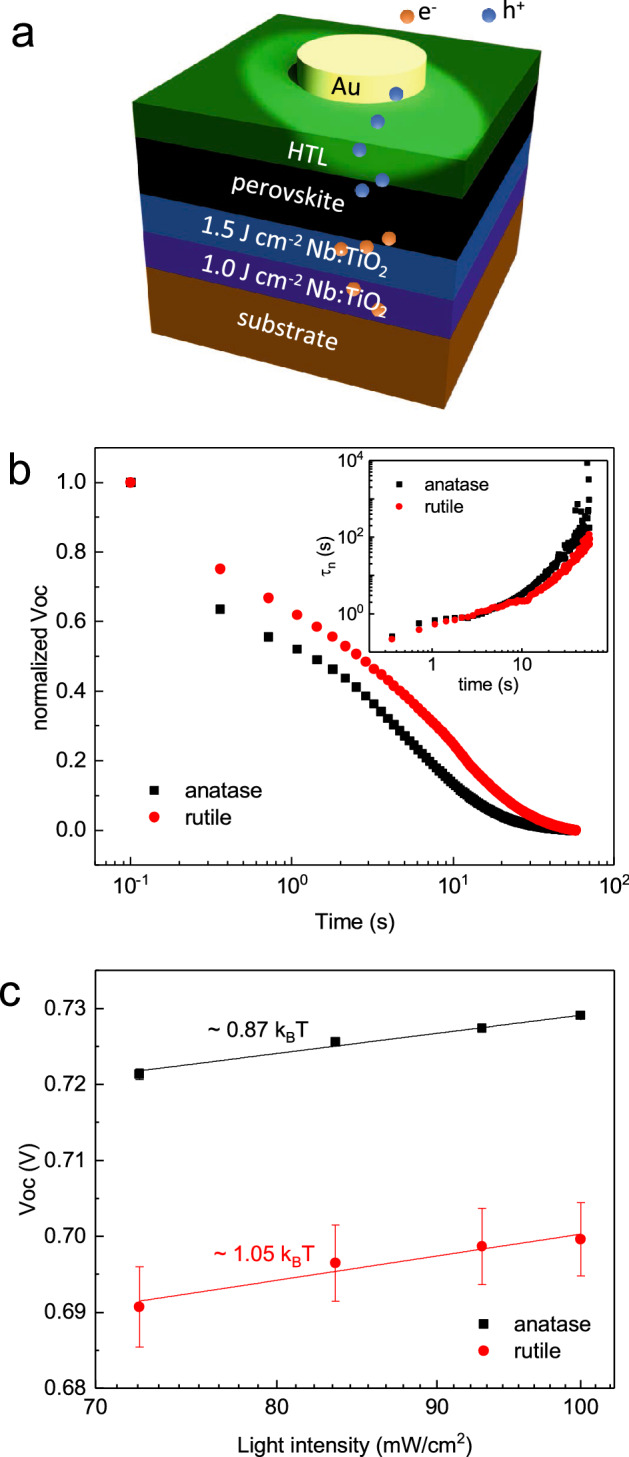
Electrical properties of the HPSC with different ETLs. (**a**) Schematic of the HPSC under illumination. (**b**) OCVD decay curves of the HPSC with different ETLs using the anatase or rutile phase on a semi-log scale. Inset of (**b**) shows the electron lifetime on a log–log scale from Eq. (2). (**c**) V_OC_ as a function of the light intensity on a semi-log scale.

To measure the PCE, Au/spiro-OMeTAD/perovskite on anatase Nb-doped TiO_2_ with a fluence of 1.5 J cm^−2^/anatase Nb-doped TiO_2_ with a fluence of 1.0 J cm^−2^/LaAlO_3_ substrate (anatase HPSC) and Au/spiro-OMeTAD/perovskite on rutile Nb-doped TiO_2_ with a fluence of 1.5 J cm^−2^/rutile Nb-doped TiO_2_ with a fluence of 1.0 J cm^−2^/γ-sapphire substrate (rutile HPSC) were prepared as shown in Fig. 4a. A 250-µm-sized Au top electrode was deposited by an e-beam evaporator with a thickness of 40 nm. The current–density voltage (*J–V*) curves are shown for the different phases of the ETL (Fig. S6, Supplementary Information). It can be observed that the HPSC with the anatase phase demonstrates a better performance compared with the HPSC with the rutile phase. Additionally, the anatase HPSC exhibits a lower series resistance and higher shunt resistance compared to that of rutile HPSC (Fig. S7, Supplementary Information). There are several reasons for the unexpectedly low PCE. First, the high resistivity of the bottom electrode (~ 10^−2^ Ω cm) compared with conventional transparent conducting oxides, such as ITO (~ 10^−4^ Ω cm)^31^, leads to high series resistance. Second, light incident on the rear contact is partially blocked by the Au electrode as shown in Fig. 4a. In addition, the electrical measurement is performed with probing contacts at the probe-station, which also blocked some of the incident light.

The open-circuit voltage (V_OC_) is an important factor not only to determine the efficiency of the solar cell but also to investigate carrier recombination properties^32,33^. To obtain reliable V_OC_ data under an open-circuit condition, we measured the V_OC_ as average readouts of a repeatedly applied current of ± 1 nA. Open-circuit voltage decay (OCVD)^33^ measurements were performed by recording the V_OC_ as a function of time (Fig. 4b). When the light illumination was switched off at t = 0, the photo-induced electrons exponentially decayed due to the recombination process corresponding to the electron lifetime. The electron lifetime can be obtained using the following equation^33^:

2 $$\tau_{n} = \frac{{ - k_{B} T}}{e}\left[ {\frac{{{\text{d}}V_{OC} }}{{{\text{d}}t}}} \right]^{ - 1} ,$$

where *k*_*B*_*T* is the thermal energy and *e* is the elementary charge. The electron lifetime derived from the OCVD results as a function of time is depicted in the inset of Fig. 4b, which shows that the anatase phase clearly demonstrates a longer electron lifetime and slower recombination kinetics than that of rutile phase, results of which are consistent with those of the TRPL.

Figure 4c shows that the V_OC_ of the anatase and rutile HPSCs varied linearly as a function of the logarithm of the light intensity and can be given by^34,35^

3 $$V_{OC} = \frac{{E_{gap} }}{q} - \frac{{k_{B} T}}{q}ln\left( {\frac{{\left( {1 - P} \right)\left( {B_{L} + B_{SRH} } \right)N_{C}^{2} }}{PG}} \right),$$

where *E*_*gap*_ is the energy gap, *P* is the dissociation probability of the excitons, *G* is the generation rate of the exciton, *Nc* is the effective density of states, and *B*_*L*_ and *B*_*SRH*_ are the Langevin recombination strength and the Shockley, Read, and Hall (SRH) recombination strength, respectively, where the Langevin and SRH recombination denote bimolecular and trap-assisted recombination, respectively. Only the generation rate *G* is proportional to the light intensity in this equation, in which the slope can be expressed in units of *k*_*B*_*T/q*. Although the effective density of states of anatase (*N*_*C,anatase*_ ~ 7.8 × 10^20^ cm^−3^)^36^ is larger than that of rutile (*N*_*C,rutile*_ ~ 2.5 × 10^19^ cm^−3^)^37^, the slope of anatase (0.87 *k*_*B*_*T/q*) is more gradual compared with that of rutile (1.05 *k*_*B*_*T/q*). Considering that trap-assisted recombination is minimized due to epitaxy growth, the lower bimolecular recombination strength in the anatase phase is in good agreement with the TRPL and OCVD results.

Also, effect of shunt resistance and RC-effect should be considered due to their substantial effect on open circuit voltage characteristics^38,39^. Low shunt resistance gives rise to rapidly decrease of OCVD signal generally within a very short time scale, such as few seconds^39^, however, their decay lasted up to several tens of seconds in this experiment. In addition, the lower shunt resistance leads to the higher slope in the V_oc_ *vs* light intensity^39^. Although the anatase phase has lower shunt resistance (Fig. S7, Supplementary Information), it shows lower slope than of the rutile phase as seen in Fig. 4c. Furthermore RC-effect due to large time constant could over-estimated carrier life time^40^. Although larger dielectric permittivity^41^ and the series resistance (Fig. S7, Supplementary Information) of the rutile phase, however, longer carrier lifetime is observed in the anatase phase as seen in the inset of Fig. 4b. Therefore, we believe that the shunt resistance and the RC-effect do not have a considerable effect on the open circuit voltage characteristics. Moreover, surface trap-assistant recombination could lower slope less than *k*_*B*_*T*/*q*^42,43^, accompanying with significantly reduce of open circuit voltage as well^44^. However, since the open circuit voltage of the anatase phase shows higher than that of the rutile phase for entire light intensities in Fig. 4c, thus we believe that the surface trap-assistant recombination has no significant influence on the interface between the halide perovskite layers and the epitaxial electron transport layers.

**Figure 5:**
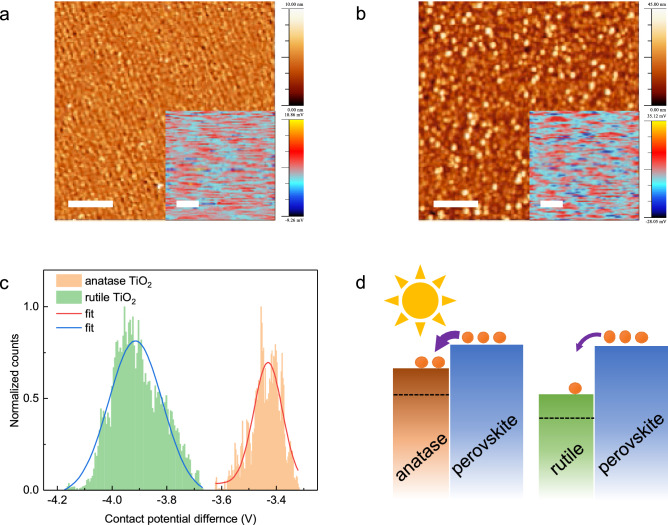
AFM and KPFM analyses. AFM topography images of (**a**) anatase and (**b**) rutile phases. Insets of (**a**) and (**b**) show the CPD maps for the corresponding topographies. Scale bar indicates 1 µm. (**c**) CPD distribution of anatase and rutile phases. Lines indicate Gaussian distribution fitting results. (**d**) Illustration of the band diagram of perovskite on the anatase and rutile phase under illumination. Only the conduction bands of the ETLs was shown to more clearly indicate the electron transporting behavior. The thickness of arrows and orange dots indicate the charge transport at the interface and the electrons, respectively.

Previous studies have demonstrated the better photo-induced activity of the anatase phase than of the rutile phase in terms of deeper bulk-to-surface carrier excitation^2^, smaller effective mass^37^, and indirect band structures^5^. Other key parameters to maintain that can increase the PCE include a small conduction band offset, particularly to improve the open circuit voltage^45^, and a smooth surface to reduce internal light scattering^46^. To investigate the surface morphology and work function difference, we measured the topography and contact potential difference (CPD) simultaneously with atomic force microscopy (AFM) and Kelvin probe force microscopy (KPFM)^47,48^. Figure 5a,b represents the surface morphology of anatase and rutile Nb-doped TiO_2_ thin films, respectively. Their root-mean-square of roughness is approximately 0.9 nm and 6.6 nm for the anatase Nb-doped TiO_2_ thin films and rutile thin films, respectively, indicating that the anatase thin film is particularly suitable for minimizing light scattering at the interfaces. The insets of Fig. 5a,b represent the mapping of the CPD of the anatase Nb-doped TiO_2_ thin films and rutile Nb-doped TiO_2_ thin films, respectively. The CPD originates from the potential difference between the tip of the cantilever and the surface of the sample. The obtained CPD distribution histogram of the anatase and rutile Nb-doped TiO_2_ thin films are shown in Fig. 5c. The measured work function difference is ~ 0.4 eV, which is consistent with previous results^49,50^. Figure 5d displays the band diagrams of different electron extraction behavior from perovskite to the anatase and rutile phase under illumination.

## Conclusions

In summary, we have deposited epitaxial anatase and rutile TiO_2_ thin films as ETL for HPSCs. The structural analysis indicates that the films’ single phase possesses high crystalline qualities. Conducting epitaxial Nb-doped TiO_2_ was prepared as a cathode by modulating the laser fluence. In comparison with the rutile phase, the anatase phase shows superior electrical and optical characteristics. The photoluminescence and open-circuit voltage analyses demonstrate that both the anatase and rutile phases show minimized trap-assisted recombination. This reduced trap-assisted recombination is a direct indication of the reduced trap density, which is highly correlated to long-term stability. In particular, the anatase phase has lower recombination properties likely due to having better band alignment and a flatter surface that results in lower light scattering at the interface. We believe that epitaxial growth-based single-phase studies not only demonstrate the surpassing properties of the anatase phase but also have the potential to significantly extend into the long-term stability through reduced extrinsic recombination in the future.

## Methods

### Epitaxial thin film preparation

 We deposited epitaxial anatase and rutile TiO_2_ thin films on LaAlO_3_ and γ-sapphire 0.5-mm-thick single-crystal substrates, respectively, using the pulsed layer deposition technique with an oxygen partial pressure of 100 mTorr at a temperature of 750 °C. The repetition of the laser was 5 Hz and 2 Hz with a laser fluence of 1 J cm^−2^ and 1.5 J cm^−2^, respectively.

### Halide Perovskite precursor preparation

 The detailed procedures are described in a study by Saliba et al.^51^ First, we prepared 1.5 M stock solutions of PbI_2_, PbBr_2_, and RbI in dimethylformamide (DMF): dimethyl sulfoxide (DMSO) with 4:1 V/V and CsI in pure DMSO. The solutions were heated at 180 °C for 10 min to be fully dissolved. Methylammonium bromide (CH_3_NH_3_Br, MABr) and formamidinium iodide (NH_2_CHNH_2_I, FAI) powders were added to the stock solutions of PbBr_2_ and PbI_2_, respectively, to obtain an amount of slightly excessive PbI_2_ with a stoichiometry of 1:1.09. Next, 0.75 mL of FAPbI_3_ and 0.15 mL of MAPbBr_3_ solutions were combined to prepare the (FAPbI_3_)_0.83_(MAPbBr_3_)_0.17_ precursor solution. As additives, 0.048 mL of CsI and 0.052 mL of RbI from the stock solutions were added.

### HPSC fabrication

 The resulting halide perovskite solution was coated onto the epitaxial anatase and rutile thin films by a sequential two-step spin-coating, in which the spin rate of the first step was 1000 rpm for 10 s with an accelerated speed of 200 rpm/s and the spin rate of the second step was 6000 rpm for 20 s with an accelerated speed of 2000 rpm/s. At the end of the 15 s during the second step, 200 µl of chlorobenzene as the antisolvent was dropped into the center of the substrate. Then, the films were moved onto a pre-heated hot plate and annealed at 100 °C for 50 min.

After cooling down to room temperature, the HTL solution was dropped during spinning on top of the thin film by spin coating at 4000 rpm for 10 s using a chlorobenzene solution containing Spiro-OMeTAD (50 mg in 0.498 mL), which included as additives 0.018 mL of 4-tert-butylpyridine, 0.010 mL of 1.8 M lithium bis(trifluoromethanesulfonyl)imide stock solution, and 0.004 mL of 0.25 M tris(2-(1H- pyrazol-1-yl)-4-tert-butylpyridine)cobalt(III) tri[bis- (trifluoromethane)sulfonimide] (FK209) stock solution.

### HPSC characterization

 The *J*–*V* curves of the HPSCs were measured using a Keithley 4200 source meter under simulated 1-sun illumination at AM 1.5G, 100 mA∙cm^–2^ (Sol3A Class AAA, Oriel, Newport) in an ambient atmosphere at room temperature. The AFM topography and KPFM measurements were performed by a n-Tracer (Nano Focus) in non-contact mode. The steady-state PL spectra and TRPL curves were collected using a commercial time-correlated single-photon counting system (FluoTime 300, PicoQuant). XPS measurements were obtained with a Thermo Scientific K-Alpha spectrometer.

## Supplementary Information

Supplementary information.
