# Supplementary material for: Reduced extrinsic recombination process in anatase and rutile TiO2 epitaxial thin films for efficient electron transport layers
Source: Sci Rep. 2021 Mar 24;11:6810. doi: 10.1038/s41598-021-86422-9 (PMC7990940; doi:10.1038/s41598-021-86422-9)
Supplement: Supplementary file 1 — Supplementary information. [file 41598_2021_86422_MOESM1_ESM.docx]

**Supplementary Information**

**Reduced extrinsic recombination process in anatase and rutile TiO_2_ epitaxial thin films for efficient electron transport layers**

**Yeon Soo Kim, Hye-Jin Jin, Hye Ri Jung, Jihyun Kim, Bich Phuong Nguyen, Juran Kim, and William Jo^*^**

Department of Physics and New and Renewable Energy Research Center (NREC), Ewha Womans University, Seoul 03760, Korea

*Correspondence and requests for materials should be addressed to W. J. (email: [wmjo@ewha.ac.kr](mailto:wmjo@ewha.ac.kr))

**Figure S1.** X-ray reflectometry measurement. (a) Normalized reflectivity of measured (black line) and fitted (red line) data of anatase TiO_2_ thin film on LaAlO_3_ substrate and (b) corresponding fast Fourier transform profile.

a

b

Below the critical angle *θ*_C_, it is assumed that the X-ray beam is completely reflected. Clear interference fringes that are well-fitted can be observed in Figure S1(a). The distance between the fringes is inversely proportional to the film thickness and can be estimated from the peak position of the Fourier transform profile. Figure S1(b) shows the fast Fourier transform profile, which was obtained using *REFLEX*.^1^


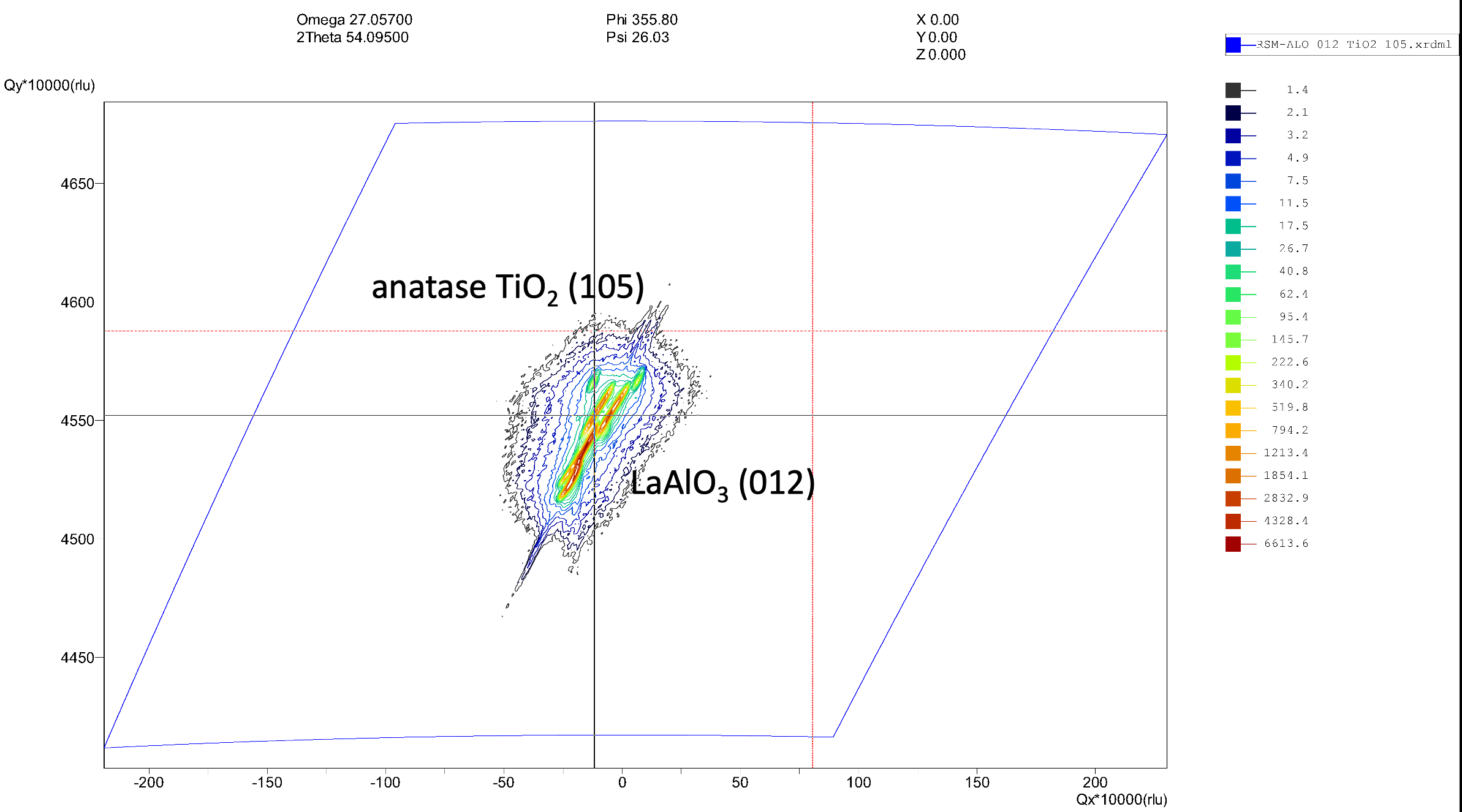

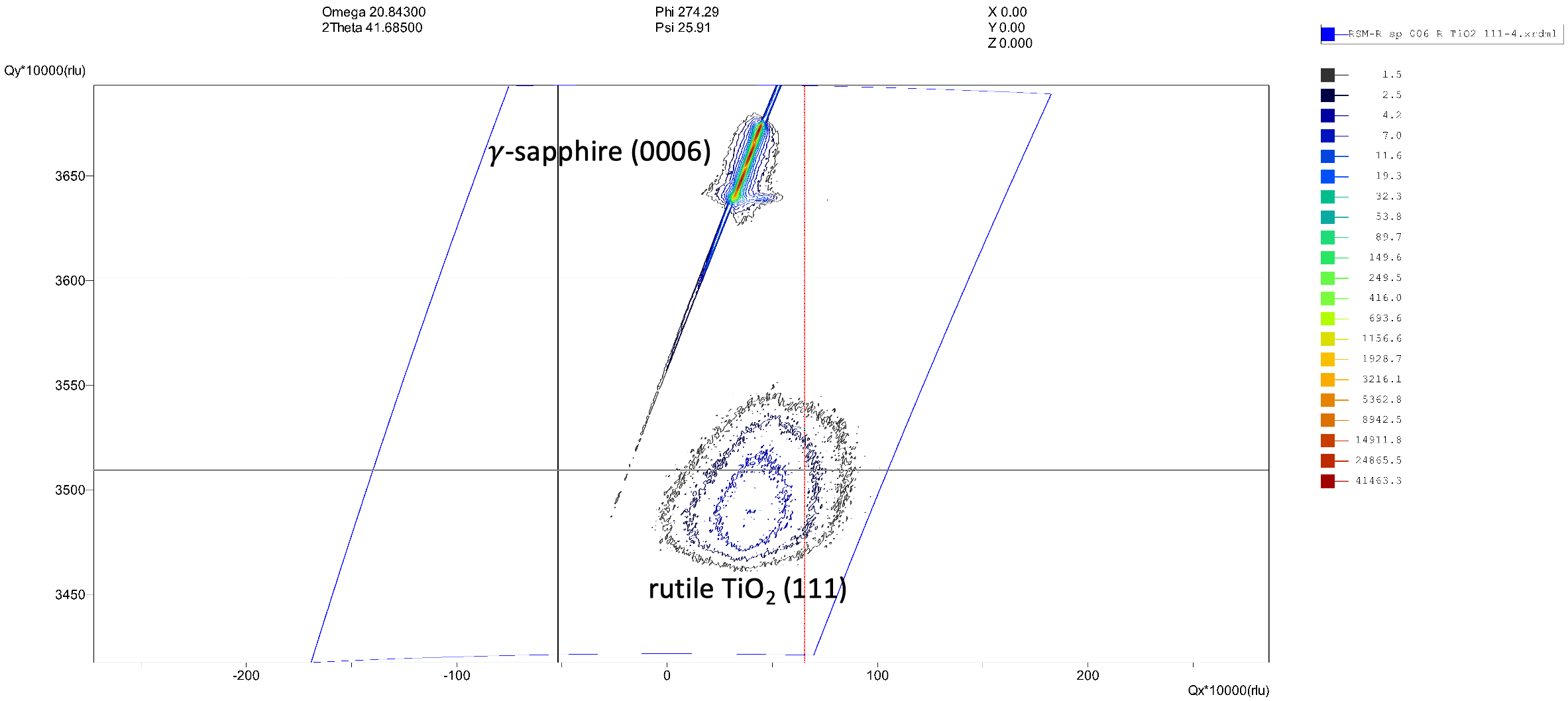


**Figure S2.** Reciprocal space mapping data of anatase TiO_2_ thin film (105) /LaAlO_3_ (012) (left) and rutile TiO_2_ thin film/γ-sapphire (right). Both films show fully lattice relaxation to in-plane direction. Due to compressive strain of anatase TiO_2_ thin films along *c*-axis, anatase (105) peak located higher Q_y_ and overlapped with LaAlO_3_ (012) peak.

**Figure S3.** X-ray diffraction data of rutile TiO_2_ thin film/γ-sapphire (black line) and rutile Nb-doped TiO_2_ thin film/γ-sapphire (red line).


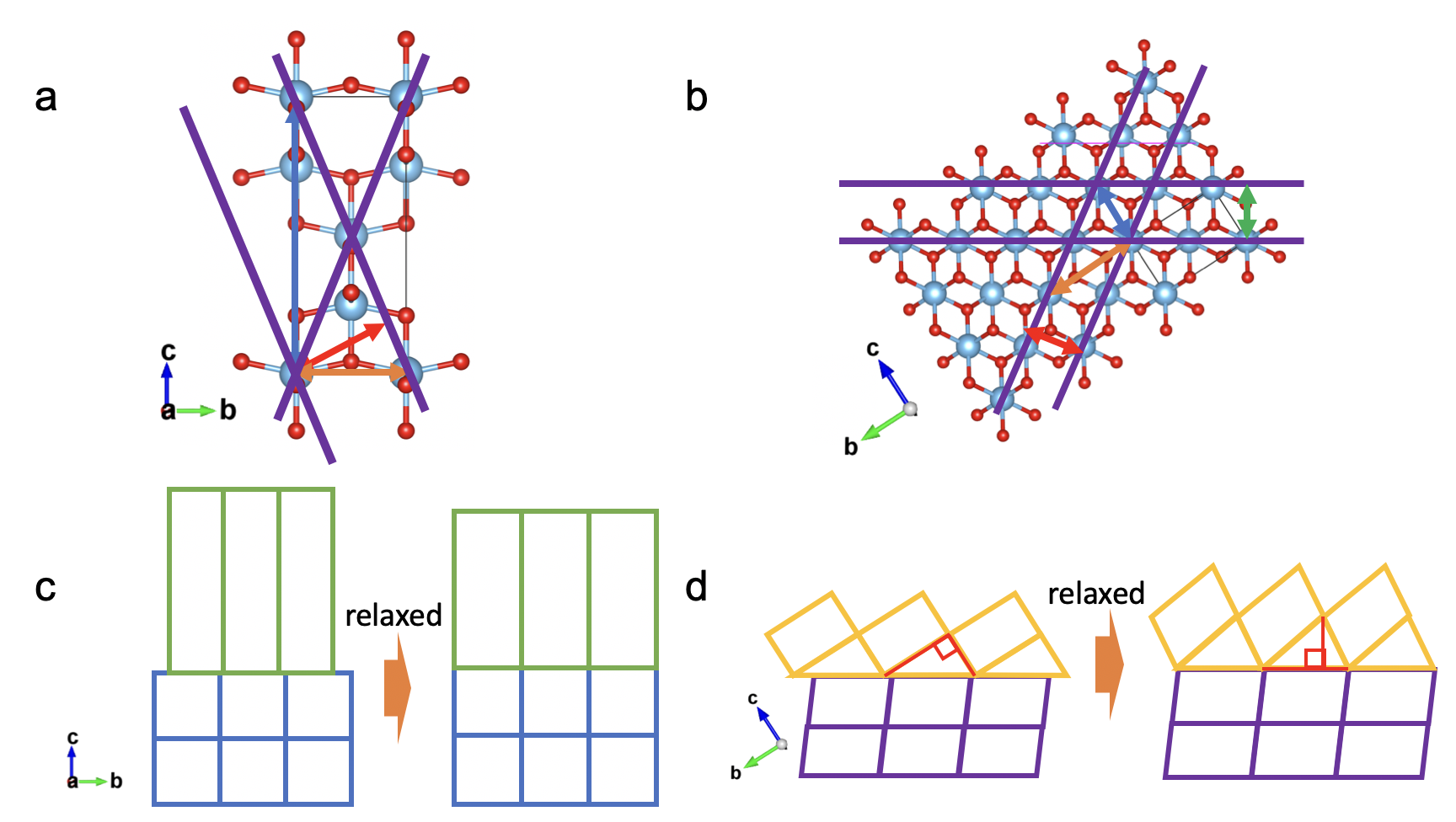


**Figure S4.** Crystal structure of bulk (a) anatase and (b) rutile phase, which were rendered using VESTA.^2^ The purple lines are taken from the TEM images. The blue, orange, red, and green arrows indicate lattice spacing for (001), (010), (011), and ($0\bar{1}1$), respectively. A schematic of lattice relaxation of the (c) anatase and (d) rutile phases on the substrates.

**Table S1.** Lattice spacings of the anatase and rutile phases obtained from the TEM images and the theoretical value of bulk and lattice relaxation corresponding to Figure S2.

|  | **Lattice spacing** | **TEM** | **Theo.**  **(bulk)** | **Theo.**  **(relaxed)** |
| --- | --- | --- | --- | --- |
| anatase | (001) | 9.077 Å | 9.514 Å | 9.089 Å |
|  | (010) | 3.928 Å | 3.785 Å | 3.872 Å |
|  | (011) | 3.571 Å | 3.517 Å | 3.562 Å |
| rutile | (001) | 3.005 Å | 2.956 Å |  |
|  | (010) | 4.594 Å | 4.594 Å |  |
|  | (011) | 2.501 Å | 2.487 Å |  |
|  | ($0\bar{1}1$) | 2.530 Å | 2.487 Å |  |

The crystal structure of bulk anatase and bulk rutile are represented in Figures S4(a) and (b). Owing to the lattice mismatch between the substrate and the thin film, the epitaxy growth results in a strong clamping effect that maintains coherency. Therefore, the lattice relaxation of the thin film occurs at the interface. The schematics of the lattice relaxation are shown in Figures S4(c) and (d). Due to the large in-plane lattice parameter of the LaAlO_3_ substrate compared with that of the anatase TiO_2_ phase, a tensile strain occurs with the anatase phase (Figure S4 (c)). In contrast, lattice distortion corresponding to a compressive strain occurs due to the smaller in-plane lattice parameter of the γ-sapphire substrate compared with that of the rutile TiO_2_ phase (Figure S4 (d)).

**Figure S5.** The peak intensity ratio of Ti^3+^/[Ti^4+^ + Ti^3+^] (red) and OH^-^/[O^2-^ + OH^-^] (blue) from the XPS spectra.


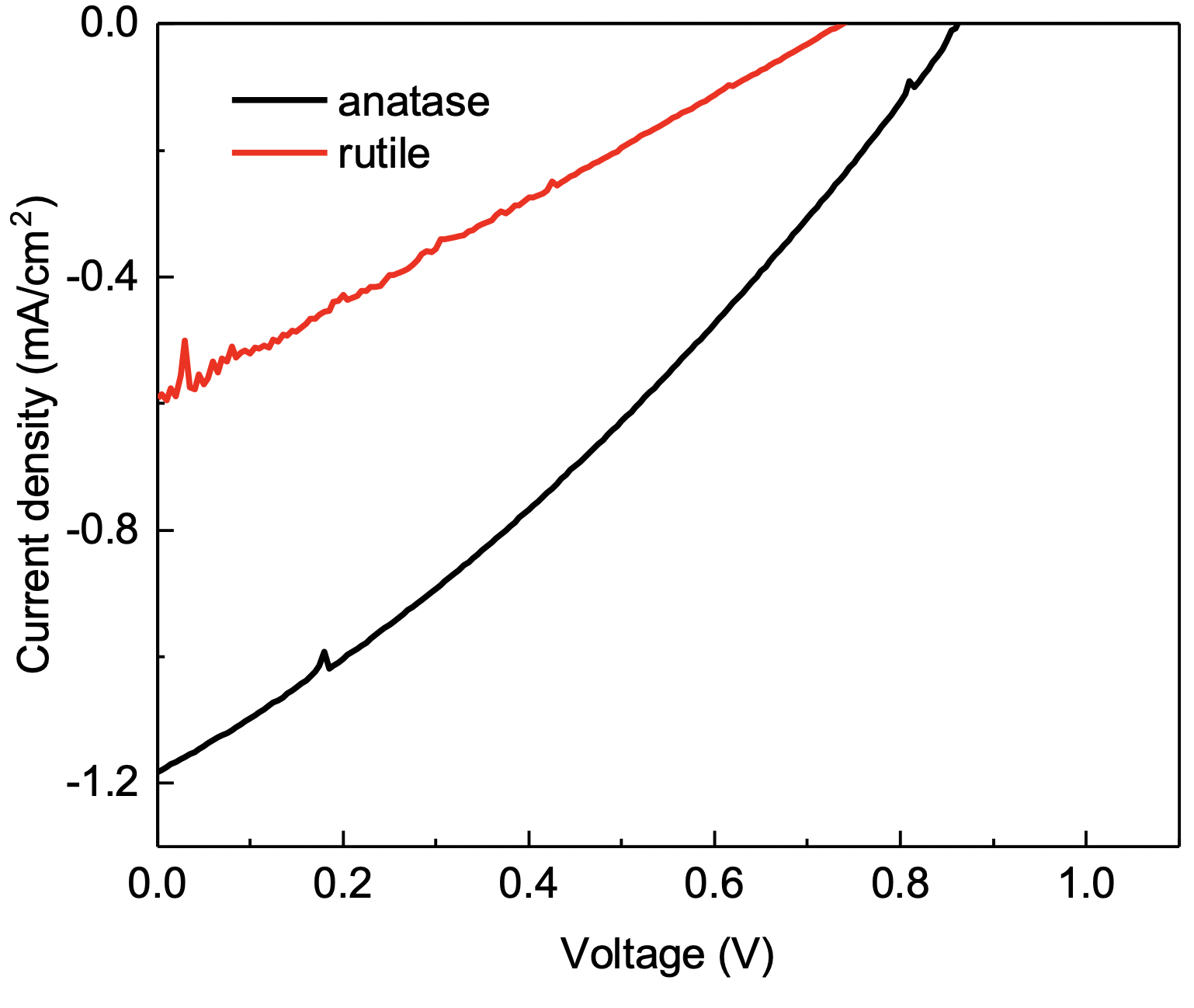


**Figure S6.** *J-V* curves of the HPSC with different ETLs using the anatase or rutile phase.

a

b

a

b

**Figure S7.** (a) Series resistance and (b) shunt resistance of anatase and rutile HPSCs. The series resistance and shunt resistance are obtained from the diode equation.^3,4^ The black and red lines serve as a guide for the eye.

**Reference**

1. Vignaud, G. & Gibaud, A. REFLEX: A program for the analysis of specular X-ray and neutron reflectivity data. *J Appl Crystallogr* **52,** 201–213 (2019).

2. Momma, K. & Izumi, F. VESTA 3 for three-dimensional visualization of crystal, volumetric and morphology data. *J Appl Crystallogr* **44,** 1272–1276 (2011).

3. Shi, J. *et al.* Hole-conductor-free perovskite organic lead iodide heterojunction thin-film solar cells: High efficiency and junction property. *Appl. Phys. Lett.* **104,** 063901 (2014).

4. Brus, V. V., Proctor, C. M., Ran, N. A. & Nguyen, T.-Q. Capacitance Spectroscopy for Quantifying Recombination Losses in Nonfullerene Small-Molecule Bulk Heterojunction Solar Cells. *Adv. Energy Mater.* **6,** 1502250–8 (2016).
